# SWISS MADE: Standardized WithIn Class Sum of Squares to Evaluate Methodologies and Dataset Elements

**DOI:** 10.1371/journal.pone.0009905

**Published:** 2010-03-26

**Authors:** Christopher R. Cabanski, Yuan Qi, Xiaoying Yin, Eric Bair, Michele C. Hayward, Cheng Fan, Jianying Li, Matthew D. Wilkerson, J. S. Marron, Charles M. Perou, D. Neil Hayes

**Affiliations:** 1 Department of Statistics and Operations Research, University of North Carolina, Chapel Hill, North Carolina, United States of America; 2 Lineberger Comprehensive Cancer Center, University of North Carolina, Chapel Hill, North Carolina, United States of America; 3 Department of Otolaryngology/Head and Neck Surgery, University of North Carolina, Chapel Hill, North Carolina, United States of America; 4 School of Dentistry, University of North Carolina, Chapel Hill, North Carolina, United States of America; 5 Department of Biostatistics, University of North Carolina, Chapel Hill, North Carolina, United States of America; 6 Department of Genetics, University of North Carolina, Chapel Hill, North Carolina, United States of America; 7 Department of Pathology and Laboratory Medicine, University of North Carolina, Chapel Hill, North Carolina, United States of America; 8 Division of Medical Oncology, Department of Internal Medicine, University of North Carolina, Chapel Hill, North Carolina, United States of America; Baylor College of Medicine, United States of America

## Abstract

Contemporary high dimensional biological assays, such as mRNA expression microarrays, regularly involve multiple data processing steps, such as experimental processing, computational processing, sample selection, or feature selection (i.e. gene selection), prior to deriving any biological conclusions. These steps can dramatically change the interpretation of an experiment. Evaluation of processing steps has received limited attention in the literature. It is not straightforward to evaluate different processing methods and investigators are often unsure of the best method. We present a simple statistical tool, Standardized WithIn class Sum of Squares (SWISS), that allows investigators to compare alternate data processing methods, such as different experimental methods, normalizations, or technologies, on a dataset in terms of how well they cluster *a priori* biological classes. SWISS uses Euclidean distance to determine which method does a better job of clustering the data elements based on *a priori* classifications. We apply SWISS to three different gene expression applications. The first application uses four different datasets to compare different experimental methods, normalizations, and gene sets. The second application, using data from the MicroArray Quality Control (MAQC) project, compares different microarray platforms. The third application compares different technologies: a single Agilent two-color microarray versus one lane of RNA-Seq. These applications give an indication of the variety of problems that SWISS can be helpful in solving. The SWISS analysis of one-color versus two-color microarrays provides investigators who use two-color arrays the opportunity to review their results in light of a single-channel analysis, with all of the associated benefits offered by this design. Analysis of the MACQ data shows differential intersite reproducibility by array platform. SWISS also shows that one lane of RNA-Seq clusters data by biological phenotypes as well as a single Agilent two-color microarray.

## Introduction

### Experimental Motivation

Suppose an investigator has a dataset that has a fixed number of samples designed to measure biological differences (such as tumor/normal) and wants to process the data, but the optimal processing method is unknown. This processing may involve background correction, normalization, sample selection, or feature/gene selection. A central question is, “Which processing method works best on a given dataset?”

There are a variety of papers in the literature which address the above question [1]–[8]. However, criteria used to compare certain processing methods are not easily applied to answer different processing problems. For example, Ritchie *et al* [9] compare background correction methods for two-color microarrays by comparing MA-plots, precision as measured by the residual standard deviation of each probe, bias and differential expression as measured by SAM regularized *t*-statistics [10]. In comparing Affymetrix microarray normalization methods, Bolstad *et al* [11] perform variance, pairwise and bias comparisons between arrays. These in-depth analyses are useful and informative. However, they can be very complex to implement and interpret. Thus, it may be unproductive for an investigator to invest sufficient time for this in every dataset, and for all aspects of experimental design. In addition, after performing these in-depth analyses, the “best method” is not always clear because many analyses do not report p-values and are instead based on subjective evaluations (such as looking at MA plots). We propose a method that is not specific to the processing method or platform under investigation and that reports a p-value which easily allows investigators to determine whether two processing methods are statistically equivalent or if one method significantly outperforms the other.

### Generalizing the Problem

Many problems can arise when trying to evaluate two processing methods or compare different platforms. For instance, the best way to compare methods/platforms is not always clear when the data are on different scales or the methods have different (unknown) distributions. Also, investigators may not be interested in measuring phenotypes, but rather measuring the elements of the phenotypes. It is also important for investigators to select the optimal method independent of the results.

Motivated by these problems, our goal is to develop a more generic approach to comparing processing methods or platforms. Our method, Standardized WithIn class Sum of Squares (SWISS), uses gene expression (Euclidean) distance to measure which processing method under investigation does a better job of clustering data into biological phenotypes (or other pre-defined classes, which could be chosen using a clustering method such as k-means or hierarchical clustering). SWISS takes a multivariate approach to determining the best processing method. It tends to down-weight noise genes (genes with little variation across all samples) while depending more on differentially expressed genes (genes with large variation between the classes). We also develop a permutation test based on the SWISS scores that allows an investigator to determine if one processing method is significantly better than another method.

Using the within class sum of squares to compare how well data are clustered has appeared before in the literature. For instance, Kaufman and Rousseeuw [12] use within class sum of squares (which they refer to as WCSS) as a tool to aid in the decision of the number of clusters that should be used for k-means clustering, and which Giancarlo *et al* [13] show to be a reasonable method for choosing k. Additionally, Calinski and Harabasz [14] proposed a method based on within and between class sum of squares that was repeatedly shown to perform well for choosing k. However, because neither method is standardized, they are only able to be used to compare the effectiveness of clustering methods when the total sum of squares is constant. Thus, they are used in choosing the best k and the best way to cluster the data, and are not able to compare the effectiveness of clustering on two different methods/platforms when the processed data given by those methods are on different scales (have different total sum of squares). To our knowledge, there are no methods currently in the literature that are able to address the variety of problems that SWISS is able to. SWISS can operate on different distance metrics. Here, we evaluate SWISS scores using Euclidean distance, which has been shown to be a reasonable way to evaluate the clustering of microarray data [15].

There are several advantages of SWISS. As previously mentioned, because we are standardizing the within class sum of squares by dividing by total sum of squares (giving a value between zero and one), SWISS can be used to compare methods that are on different scales. For example, different scales can arise from differing normalization methods or when comparing different platforms. Another advantage is that SWISS can be used to compare methods that have different dimensions. This can be useful when comparing the same biological samples, but using two different gene sets. Finally, because the permutation test reports a p-value, we are able to decide which processing method is preferred without relying on subjective evaluation.

### Experimental Application I: Two-Color versus One-Color Microarrays

We will use SWISS to evaluate the one-color versus two-color microarray problem. Two-color gene expression array assays are among the most common genomic profiling tools currently in use [16]–[19]. Two-color array technologies rely on labeling two samples (such as tumor vs. normal or experimental vs. reference) with different fluorochromes (such as Cy3 and Cy5) followed by co-hybridization to the same chip-based assay [20]. The most compelling of reported incentives for the co-hybridization strategy has been to control for technical variability in array manufacturing [21]. Considering relative fluorescence (such as a log-ratio), particularly to a common reference such as a cell line reference hybridized on the same array, provides a robust normalization technique to control for such manufacturing variability [22]. A two-color array with a common reference such as a cell line will be referred to as a “reference design”. A one-color array or a two-color array using only one signal channel will be referred to as a “single-channel design”.

The reference design, while powerful, has its disadvantages [16]; notably, 50% of the measurements in a reference design experiment are solely for normalization purposes representing both significant financial and opportunity costs. Additionally, there is an effective doubling in measurement error by the reference design because every ratio includes error contributions from both experimental and reference channels [16], [19]. Furthermore, genes that are biologically absent or expressed at very low levels in the reference sample are sometimes excluded from consideration even if present at high levels in the experimental sample, which likely reduces the information content of the experiment. In contrast to the two-color arrays, one-color arrays do not rely on experimental normalization such as that described for the reference design, but rather computation techniques to normalize fluorescence intensities across arrays. Historically, the distinction between the one and two-color platforms has been viewed primarily in terms of the technology underlying the manufacturing and experimental protocols of the array platform [20].

There are many aspects to comparing one-color versus two-color arrays. First, there is the underlying experimental question of whether it is more advantageous to use a one-color or two-color array. Second, there are questions of which normalization should be used when comparing different platforms. Finally, there is the decision of which samples and which genes should be included in the analysis. We will address the above issues using our SWISS method.

### Experimental Application II: Direct Comparison of Commercial Microarray Platforms

The MicroArray Quality Control (MAQC) project was initiated to address concerns over the reliability of microarrays. In this project, gene expression levels were measured from two high-quality distinct RNA samples on seven microarray platforms (although here we only consider Affymetrix and Agilent). Each microarray platform was deployed at three independent test sites and five replicates were assayed at each site. This experimental design and the resulting datasets provide a unique opportunity to assess the repeatability of gene expression microarray data within a specific site, the reproducibility across multiple sites, and the comparability across multiple platforms [23].

We will use SWISS to address some of the issues raised by the MAQC project. Specifically, we will compare one-color Agilent microarrays, two-color Agilent microarrays, and Affymetrix microarrays by measuring how well replicates of the same samples cluster together. We will also compare Affymetrix pre-processing methods RMA [24] and the Affymetrix Micro Array Suite 5.0 (MAS 5.0) [25].

### Experimental Application III: RNA-Seq versus Gene Expression Microarrays

Microarrays have been the technology of choice for large scale studies of gene expression. However, array technology has its limitations. First, each microarray can only provide information about the genes that are included on the array. Second, there are multiple sources of variability such as differences in arrays, dye labeling, efficiency in reverse transcription, and hybridization [26]. Additionally, hybridization results from one sample may not provide a reliable measure of the relative expression of different transcripts [27]. Sequencing based approaches, such as RNA-Seq, have the ability to overcome these limitations. We will use SWISS to compare a single Agilent microarray to one lane of RNA-Seq.

## Materials and Methods

### Standardized WithIn class Sum of Squares (SWISS)

Let 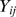 be a *d*-dimensional vector of covariates (such as gene expression) of the *j* ^th^ observation 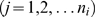 from the *i* ^th^ class/phenotype 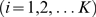. Let 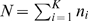 be the total sample size, 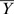 be the *d*-dimensional overall mean of all *N* samples, and 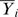 the mean of class *i*. Following classical statistical ANalysis Of VAriance (ANOVA) ideas, the Total Sum of Squares (SST) is defined to be
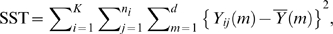
and the Total WithIn class Sum of Squares (Total WISS) is
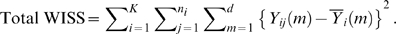



Then the Standardized WithIn class Sum of Squares (SWISS), which is the proportion of variation unexplained by clustering, is defined as
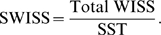



**Figure 1:**
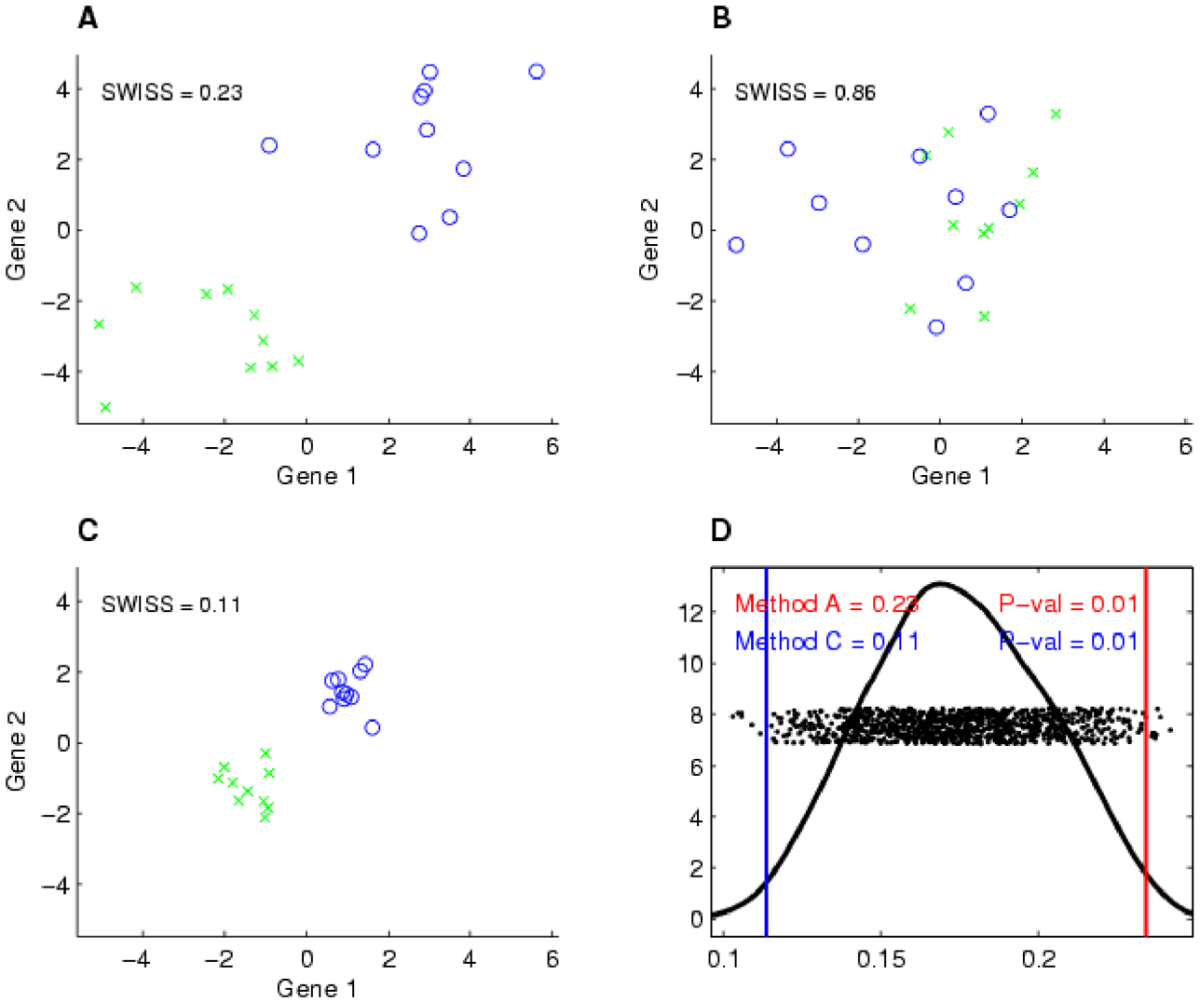
Toy example demonstrating how SWISS measures clustering. Two-dimensional toy example, with the same axes in plots A–C. The two classes are distinguished by different colors and symbols. Suppose that the same dataset has been processed using three different methods, with the processed data shown in A–C. This toy example demonstrates that data that are clustered better (A and C) have a lower SWISS score than data where there is not much separation between classes (B). This also shows that SWISS scores can be compared even when the data are on different scales (A vs. C). Plot D shows the SWISS permutation test of the data shown in plots A and C. This plot shows the distribution of the permuted population of SWISS scores (black dots), summarized by a smooth histogram (black curve), along with the SWISS scores of Method A (red vertical line) and Method B (blue vertical line). The SWISS scores and corresponding empirical p-values are also reported. Because both p-values are less than 0.05, we conclude that the processing method shown in C is significantly better than the processing method shown in A.

Suppose we have two processing methods (such as normalization techniques) that we are interested in comparing on the same dataset with pre-defined classes or phenotypes (such as tumor/normal). We consider Method A to be “better” than Method B if each of the classes of Method A have “tighter” clusters and/or have larger distances between the classes than Method B. When this occurs, SWISS will report a lower score for Method A. This is shown by a 2-dimensional toy example with two phenotypes in Figure 1. The processing method of Figure 1A (which we will refer to as Method A) is “better” than the method of Figure 1B (Method B) because the two classes (denoted by different colors and symbols) have better separation, and hence, there is a lower SWISS score. Notice that the axes are the same for plots A–C in Figure 1. When comparing the clustering of the methods shown in Figures 1A and 1C (Methods A and C), we cannot simply compare within class sum of squares because the datasets are on different scales. However, once we standardize the within class sum of squares, the SWISS scores have the same scale and are comparable. Therefore, we are still able to compare SWISS scores between plots A–C. Because the SWISS score of Method C is lower than the SWISS score of Method A, we can conclude that Method C is preferred over Method A.

However, suppose the investigator has a preference for using Method A. For example, Method A may be easier to implement, or may be more cost effective, as is the case when considering whether one-color arrays perform as well as two-color arrays. To answer the question of whether the difference between the SWISS scores of Methods A and C is statistically significant, we developed a permutation test based on SWISS. This permutation test is described in detail in Text S1 in the supporting information section. Two p-values will be reported (one for each method), and we will conclude that Method C is significantly better than Method A if the SWISS score for Method C is smaller than for Method A, and both reported p-values are less than 0.05.

Figure 1D shows the SWISS permutation test comparing Methods A and C. The SWISS scores of both methods are shown at the top left of the plot. The x-axis shows the range of SWISS scores, and the red and blue vertical lines show the SWISS scores of Method A and Method C, respectively. The black dots show the distribution of the permuted population of SWISS scores (with random heights), and the black line shows a smooth histogram of these black dots. The p-values are calculated by taking the proportion of permuted SWISS scores to the left of Method C's SWISS score (or the smaller SWISS score of the two methods being compared), and the proportion to the right of Method A's SWISS score (the larger SWISS score of the two methods). Since both p-values are less than 0.05 and Method C has a smaller SWISS score, we conclude that Method C is significantly better than Method A.

R and Matlab code for calculating SWISS and performing the corresponding permutation test are available at http://cancer.med.unc.edu/nhayes/pubs.html.

### Microarray Experiments, Data Collection and Processing: Experimental Application I

#### Description of the four cases used in Experimental Application I

We analyzed four cases of microarray experiments likely to represent the diversity of data presented in a typical microarray-intensive laboratory (Table 1). The cases included two different technologies; i) an early generation spotted cDNA array (dataset I) and ii) inkjet-printed long oligonucleotide arrays (datasets II–IV). Within the inkjet technology we included an older 22K Agilent oligonucleotide array platform (dataset II), and one of the company's most current platforms, the 4×44K Agilent oligonucleotide array (dataset III). Experiments on all three platforms were done using a reference design approach. The signal intensities from the Cy5-labeled experimental channel were taken as the estimation of single-channel design signals. In addition, we also carried out an experiment of self-self hybridization on the 4×44K oligonucleotide array (dataset IV).

**Table 1:** Description of the datasets/cases used in this study.

Experimental Application	Dataset	Tumor Type	Array Platform	Hybridization Method	Number of Arrays Used	Phenotype	Reference
I	I	Breast cancer	Spotted cDNA arrays (svcc-8k)	Reference design	39	Estrogen receptor (ER) status	[29]
	II	Breast cancer	Agilent 22K custom oligonucleotide arrays	Reference design	52	Estrogen receptor (ER) status	[28]
	III^a^	HNSCC^c^	Agilent 4×44K oligonucleotide arrays	Reference design	16	Tumor/Normal	
	IV^b^	HNSCC^c^	Agilent 4×44K oligonucleotide arrays	Self hybridization	16	Tumor/Normal	
II	V (Affymetrix)	UHRR and HBRR^d^	Affymetrix HG-U133 Plus 2.0 Gene Chip	Reference design	40	Samples A-D^e^	[23]
	VI (Agilent one-color)	UHRR and HBRR^d^	Agilent Whole Human Genome Oligo Microarray, G4112A	One-color design	20	Samples A-D^e^	[23]
	VII (Agilent two-color)	UHRR and HBRR^d^	Agilent Whole Human Genome Oligo Microarray, G4112A	Two-color design	10	Samples A,B^e^	[23]
III	VIII (Microarray)	Breast cancer	UNC custom Agilent two-color microarray	Reference design	8	Basal/Luminal	
	IX (RNA-Seq)	Breast cancer	Illumina Genome Analyzer II	RPKM normalized sequence tags	8	Basal/Luminal	

a,b Datasets III and IV contain the same clinical samples that were hybridized using different array designs.

c Head and Neck Squamous Cell Carcinoma.

d Universal Human Reference RNA (UHRR) and Human Brain Reference RNA (HBRR).

e Sample A = 100% UHRR; Sample B = 100% HBRR; Sample C = 75% UHRR, 25% HBRR; Sample D = 25% UHRR, 75% HBRR.

For the purpose of compatibility, both datasets I and II contained breast cancer samples and both datasets III and IV contained head and neck squamous cell carcinoma (HNSCC) samples. These sample sets were selected based on the availability of distinct phenotypes of approximately equal prevalence. The phenotypes were estrogen receptor positivity/negativity for the breast cancer samples and tumor/normal for the head and neck cancer samples. Datasets I-II were existing data previously reported [28], [29] and datasets III and IV were generated for this study.

#### Data collection

The gene expression data of two breast cancer datasets (I [29] and II [28]) were obtained from the UNC Microarray Database (https://genome.unc.edu/). Dataset II is also available under the GEO accession number GSE1992. The image files of dataset I were analyzed by using a ScanArray 3000 (General Scanning, Watertown, MA) or a GenePix 4000 (Axon Instruments, Foster City, CA) scanner and the primary data tables and the image files were originally stored in the Stanford Microarray Database (http://smd.stanford.edu//) [29]. The image files of dataset II were analyzed with GenePix Pro 4.1 [28]. The net-mean signals of Cy3 and Cy5 channels for datasets I and II were loaded into the UNC Microarray Database where a loess normalization procedure was performed to adjust between the two channels. Log-ratios of the Cy5-labeled (experimental sample) over the Cy3-labeled (reference sample) signals were obtained as gene expression measures for the reference design. The signal intensities from the Cy5-labeled experimental channel were obtained as the gene expression measure of the single-channel design method (datasets I and II).

#### Microarray experiments

As novel experiments in this study, we selected eight HNSCC tumor samples and eight normal tonsil samples collected on an IRB-approved protocol from patients treated at our institution. RNA isolation and microarray protocols were carried out as described in Hu *et al* [30]. Agilent Feature Extraction (FE) software [31] was used to analyze image files, extract signals and flag unreliable probes. These new data have been deposited into the Gene Expression Omnibus (GEO) under the accession number of GSE13398 and GSE13397.

#### Reference and Single-channel design data processing

Prior to log-ratio transformation, signal intensities of Cy5-labeled experimental channel and Cy3-labeled reference channel were background corrected and loess normalized between the two channels for dye-bias correction [32]. The normexp +offset method described in Ritchie *et al* [9] for background correction was implemented using the R package *limma* [33]. All features on the arrays were included. The background-corrected data then underwent a within array loess normalization and the log-ratio values of the normalized data were used as the signals of the reference design. The background-corrected experimental channel data were logarithm-transformed and then loess normalized across arrays. The normalized values were used as the signals of the single-channel design.

In our lab, as in many labs, Cy5 has historically been used for the experimental channel and Cy3 for the reference channel. Therefore, in these retrospective analyses, Cy5, by default, is the single-channel signal. Many investigators will be familiar with research suggesting that Cy3 is preferable due to its more favorable stability properties and this should be considered for any single-channel experiments planned as a result of the retrospective analysis [34].

#### Cluster Analysis

We first filtered the datasets down to approximately 10% of total genes (800 genes for dataset I, 2000 for dataset II, and 4000 for dataset III) based on Median Absolute Deviation (MAD). We then clustered the datasets using Consensus clustering [35] based on Pearson correlation distances with the ConsensusClusterPlus [36] function from BioConductor.

### Microarray Experiments, Data Collection and Processing: Experimental Application II

#### Data collection and processing

The Agilent and Affymetrix array data (datasets V – VII) were obtained from the MAQC website (http://edkb.fda.gov/MAQC/MainStudy/upload/). Four different samples were assayed on these platforms. The two RNA sample types used were a Universal Human Reference RNA (UHRR) from Stratagene and a Human Brain Reference RNA (HBRR) from Ambion. Sample A was 100% UHRR; Sample B 100% HBRR; Sample C 75% UHRR and 25% HBRR; Sample D 25% UHRR and 75% HBRR. The two-color Agilent arrays only used samples A and B.

The Affymetrix experiments (dataset V) were carried out at six separate test sites (we only use data from the first two test sites) with each site processing five replicate assays on each of the four samples for a total of 40 microarrays. The Agilent one-color experiments (dataset VI) were carried out at three separate test sites (we only use test site 2) with each site processing five replicate assays on each of the four samples for a total of 20 microarrays. The Agilent two-color experiments (dataset VII) were carried out at three separate test sites (we only use test site 2) with each site processing five replicate arrays on each of the two samples (A and B) for a total of 10 microarrays. For more information about the experiments, see Shi *et al* [23].

For dataset VI, we obtained the normalized data, which was transformed by setting all measurements less than 5.0 to 5.0. All data points were median scaled to 1 using the median signal intensity value for data points labeled as present. For dataset VII, we obtained the normalized log ratio data, which defined the log ratio as CH1/CH2 (Cy3/Cy5) where the Cy3 and Cy5 channel intensities were background-subtracted. Data were extracted using Agilent's Feature Extraction software, version 8.5. Only MAQC samples A and B were used in Agilent two-color experiments.

For dataset V, we obtained both the normalized and raw CEL files. For the comparison of Affymetrix and Agilent platforms, we used the normalized data from test site 2, which was normalized using PLIER [37]. An offset value of 16 was then added to each probeset-level data point. For the analysis of different Affymetrix pre-processing methods, the raw CEL file data from test site 1 were then analyzed with BioConductor to generate probeset level data using the rma and mas5 functions in R [38]. Probe-level data were first quantile normalized before applying each function.

### Microarray Experiments, Data Collection and Processing: Experimental Application III

#### Microarray data quantification and processing

Microarray experiments (dataset VIII) were performed with custom UNC Agilent two-channel microarrays. After hybridization, the arrays were scanned by Axon GenePix 4000B scanner (Axon Instruments, Foster City, CA). The images were analyzed using Gene Pix Pro 5.0 software (Axon Instruments, Foster City, CA). These data have been deposited into the Gene Expression Omnibus (GEO) under the accession number of GSE20234.

Gene expression values were quantified by the two based log ratio of red channel intensity (mean) vs. green channel intensity (mean), followed by loess normalization to remove the intensity-dependent dye bias [32]. We used UNC Microarray Database (https://genome.unc.edu/) to perform the filtering and pre-processing. The data matrix was gene median centered and sample standardized. Missing data were imputed with 10-nearest-neighbor imputation [39]. If the intensity of one probe in one array is less than 10 in the green channel or in the red channel, the expression of that probe in that array is excluded for further analysis. If a probe has more than 35% of expression values missing in all the arrays (due to low intensities or bad flags), the expression of that probe for all the arrays is excluded for the further analysis.

#### RNA-Seq data quantification and processing

mRNA was prepared from each experimental sample and the standard RNA-Seq sequencing process (dataset IX) was carried out with Illumina Genome Analyzer II. Raw short read (36 bp) sequence tags were pre-filtered according to manufacture recommended error rate. Pre-processed short read sequences were aligned using MAQ [40] to human refseq database [41] based on NCBI build 36.1. Up to two mismatches were allowed in the alignment. RPKM [42] was computed for each human transcript (existed in human refseq database as of February 18^th^, 2009) on the isoform level using the equation RPKM  = 10^9^× C/(NL), where: C is the number of reads that mapped to a transcript, N is total number of mappable reads in the experiment, and L is the length of the transcript. The average of RPKM for all isoforms within a gene locus was computed and used to represent the quantity of the genes expressed in the cell. Logarithm (base 2) transformation was applied on each RPKM value on the gene level. A link to this data has been provided in the Gene Expression Omnibus (GEO) under the accession number of GSE20234.

#### Common gene list

The 1541 genes used in this study came from four published intrinsic gene lists [28], [29], [43], [44] that were also present in both datasets VIII and IX.

## Results

### Experimental Application I: Two-Color versus One-Color Microarrays

#### Comparison of Single Channel Normalization Methods

**Figure 2:**
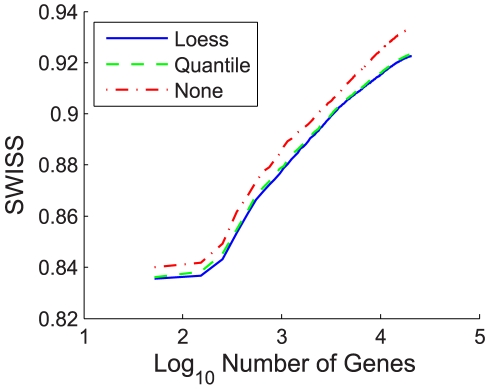
Normalization of single channel design, dataset II. Comparison of SWISS scores of three different normalization techniques for the single channel of dataset II. The number of genes was varied, as shown by the x-axis. Genes were filtered for each normalization method based on gene variation, keeping the genes with the largest variation. The normalization techniques being compared are loess (solid blue), quantile (dashed green), and no normalization (dot-dashed red). This shows that for each fixed number of genes, quantile and loess normalization are both superior to no normalization, and that loess normalization performs slightly better than quantile normalization.

SWISS can be applied to compare different normalization techniques on a dataset. We compared loess and quantile normalizations along with the raw expression data for the single channel design after performing background correction. The results of the analysis for dataset II are shown in Figure 2, where SWISS scores were calculated for each normalization method. We varied the number of genes that were filtered in our analysis. For each normalization method, filtering was based on gene variance across arrays. It is possible that for a fixed number of genes, different genes were compared across different normalization methods since normalization may have affected gene variation across arrays. It can be seen that for each fixed number of genes, quantile and loess normalization were both superior to the raw data (because they have lower SWISS scores), and that loess normalization performed slightly better than quantile normalization.

This does not show that loess normalization is the optimal normalization method for Agilent single channel array data. There are other single channel normalization methods not considered here, such as normalization based on principle component analysis [45]. However, based on the normalization methods we considered in this analysis and in the absence of an obvious standard, we decided to normalize the single channel data using loess normalization for the rest of our analyses in Experimental Application I.

#### Comparison of Reference and Single Channel Designs: Experimental Normalization

**Figure 3:**
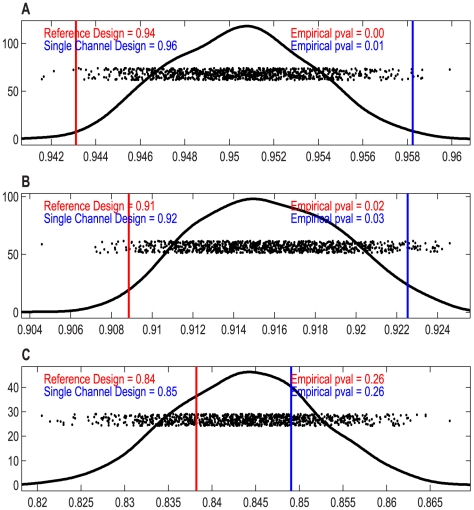
SWISS permutation test results, datasets I–III. SWISS hypothesis test results for datasets I–III (A–C). Each plot shows the distribution of the permuted population of SWISS scores (black dots), summarized by a smooth histogram (black curve), along with the SWISS scores of the reference design (red vertical line) and single channel design (blue vertical line). When both p-values are less than 0.05 (as in A and B), we conclude that the method with the smaller SWISS score (the reference design in A and B) is significantly better than the other method (the single channel design). However, if either p-value is greater than 0.05 (as in C), we conclude that there is no significant difference between the reference and single channel designs.

For datasets I – III, we performed our SWISS permutation test on the full gene set to test whether there was a significant difference in the clustering capabilities of the reference design versus the single channel design. If both reported p-values were less than 0.05, we concluded that the design with the lower SWISS was significantly better. Otherwise, we concluded that there was no significant difference between the reference and single channel designs. Figure 3 shows the results of the test.

For dataset I, we see that the reference design and single channel design have p-values of 0 and 0.01, respectively. Because the reference design has a smaller SWISS score of 0.94, we conclude that the reference design is significantly better at clustering the data of different ER status (positive vs. negative) than the single channel design. This is not surprising because dataset I used older generation spotted cDNA arrays. Thus, the two-color reference design is absolutely necessary in this case.

For dataset II, which used Agilent 22K custom oligonucleotide arrays, the reference design has a lower SWISS score of 0.91. Its p-value is 0.02, and the single channel design has p-value 0.03. Because both p-values are less than 0.05, we conclude that the reference design is significantly better than the single channel. However, both p-values are larger than the p-values for dataset I. Therefore, the difference between the reference and single channel designs is not as strongly significant for dataset II as for dataset I.

Dataset III used Agilent 4×44K oligonucleotide arrays. Because the p-values of the reference and single channel designs are both 0.26, we conclude that there is no significant advantage to using the reference design over the single channel design. Thus, we confirmed results suggested by other investigators that high-quality commercial two-color arrays are available that do not benefit in a significant manner from the normalization offered by a reference channel [46].

An important question regards the biological significance of a difference between SWISS scores. The answer will differ for each dataset, but one possible solution to this question is to introduce a perfect-feature gene. For each class, find the average of each gene across all samples within the specified class. Each sample in the specified class is assigned the maximum of these gene averages for its value of the perfect-feature gene, and each sample not in the specified class is assigned the minimum of the averages. There are as many perfect-feature genes as classes. Add all of the perfect-feature genes to the dataset then recalculate the SWISS score (which we will call the perfect-feature enhanced SWISS score). A perfect-gene approaches the smallest biologic quantity for which there is clearly a biologic interest and we can calculate the impact of adding such a feature to the dataset on the SWISS score. We can then use the SWISS score with and without a perfect-feature gene to assess the biologic significance relative to any statistically significant comparisons observed in an experiment. For example, for dataset I, the original SWISS scores of the single channel and reference designs are 0.958 and 0.943, respectively (shown in Figure 3A). The perfect-feature enhanced SWISS score of the single channel design is 0.954. Because the SWISS score of the reference design is smaller than the perfect-feature enhanced SWISS score of the single channel, we conclude that the difference in SWISS scores between the single channel and reference designs of dataset I is not only statistically significant but also biologically meaningful. We do caution that this perfect-feature gene approach is sensitive to the amount and type of gene filtering.

In each of the previous analyses, the classes used were “true” phenotype classes. We were also interested in seeing how using unsupervised methods to determine classes would change the results. We applied standard hierarchical clustering techniques. Supplementary Table S1 shows the results of the clustering and Table S2 shows the SWISS scores of datasets I – III, for both the phenotype and the Consensus clustering classifications. The SWISS scores, when using the Consensus clustering classifications, were always smaller than the SWISS scores based on phenotype classification. This is not surprising; we expect that Consensus clustering would classify samples close to each other in gene expression space as SWISS is designed to measure. For both the single channel and reference designs for datasets I and III, the difference between phenotype and clustering SWISS scores is not significant. However, for both the single channel and reference designs for dataset II, Consensus clustering gives a significantly lower SWISS score than the phenotype SWISS score. This provides evidence that either some of the breast cancer samples of dataset II may have been mislabeled with respect to their ER status or that a two class unsupervised clustering does not adequately capture the biology of ER status.

#### Feature Selection: Evaluating the Effect of Filtering

**Figure 4:**
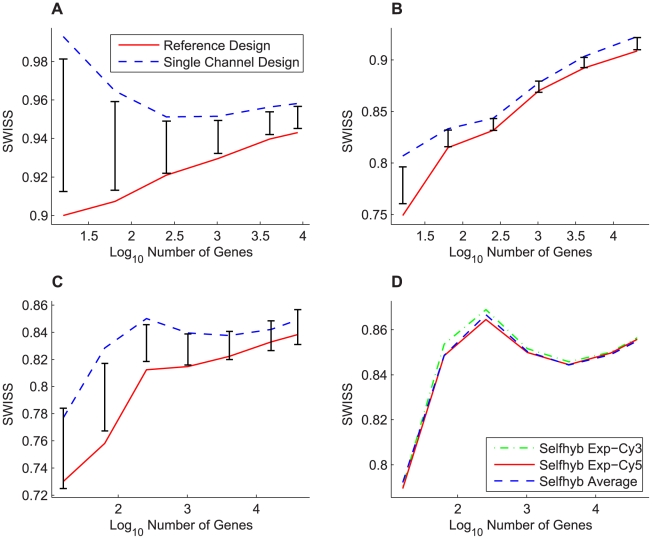
Effect of filtering genes by variance, datasets I–IV. SWISS scores for the reference design (solid red) and single channel design (dashed blue) along with corresponding 90% confidence intervals (black bars) calculated from the SWISS permutation test are shown for datasets I – III (A – C). The SWISS scores for the self-self hybridization Exp-Cy3 channel (dot-dashed green), Exp-Cy5 channel (solid red), and the average of the two self-self hybridization channels (dashed blue) are shown for dataset IV (D). In A (dataset I), the reference design is always significantly better than the single channel design (because the black bars are always inside the blue and red curves). However, in B and C (datasets II and III), there are certain gene sets where there is a significant difference between the two designs and other gene sets where there is no significant difference. In D, there is very little difference between each of the two experimental channels and the average of the two channels.

For our next analysis, we used SWISS to evaluate the effect of different types of and amounts of gene filtering. For each dataset (I – IV), we filtered the gene set by keeping the genes with the largest variances. We calculated SWISS scores starting with only 16 genes and ending with all genes included. Note that for each dataset, the gene lists of the reference and single channel designs were decided independently, and thus the gene sets may not be identical. For dataset IV, we calculated SWISS scores for the self-self hybridization experimental Cy3 and Cy5 channels, along with the average of those two channels. Figure 4 shows the results. For datasets I – III, we also show the 90% confidence interval (black bars) from the SWISS hypothesis test.

For dataset I (Figure 4A), we see that the reference design always has a lower SWISS score, and that the reference design is always statistically better than the single channel design (because the red and blue curves always lie outside of the 90% confidence interval). For dataset II (Figure 4B), even though the reference channel always has a lower SWISS score than the single channel design, it is not always significantly better. In fact, the two methods are almost statistically equivalent once we include at least 256 genes. Therefore, as long as more than 256 genes are used, the single channel design is as effective as the reference design. For dataset III (Figure 4C), the reference and single channel designs are statistically equivalent at the beginning (for 16 genes) and end (for at least 256 genes). However, there are gene filterings where the reference design becomes statistically better than the single channel design. This effect demonstrates that the selected gene set plays a significant role in comparing the performance of the single channel and reference designs.

For dataset IV (Figure 4D), the self-self hybridization Cy3 channel, Cy5 channel, and average of the two channels appear to perform equivalently in terms of SWISS scores. The SWISS permutation test can only be used to test for a significant difference between two methods, and here we are trying to compare three different methods. We did perform the permutation test on all pairs and filterings, and all of these tests returned insignificant p-values. Therefore, there is no significant difference between either of the single channels or the average of the two channels. For this reason, we did not include confidence intervals in Figure 4D. However, we notice that once we include about 1000 genes, the average of the two channels has a slightly lower SWISS score, although not significantly lower. When including all genes in our analysis, the SWISS scores for the Cy3 channel, Cy5 channel, and average of the two channels are 0.8564, 0.8558, and 0.8551, respectively. This analysis suggests that there may be some small potential for improvement over the single channel design by measuring the same biological sample in both the Cy3 and Cy5 channels and combining the data in some way. This may be a reasonable method for improving data quality without increasing costs, as this experimental set-up requires the same number of arrays as the reference design.

Notice that in Figure 4B, as the number of genes increases, the SWISS scores also increase. This suggests that the lower variable genes contain little useful phenotypic information. The curves in Figures 4C and 4D are similar to each other in shape, where they begin by increasing, then decrease for a while, then increase again at the end. This suggests that there are a small number of genes that do a reasonable job of reflecting the difference in phenotypes. Then, as we add up to 256 genes, the SWISS scores increase, which is consistent with these genes adding more noise than phenotypic information. The decrease in the middle of the curves could be a result of additional genes that can be added to the gene set that increase the distance between phenotypes. The final increase is most likely due to adding genes that are pure noise to the analysis. The curves in Figure 4A have a distinct shape, with one curve always increasing, and the other curve almost always decreasing. Our next analysis investigates why these two curves have such different shapes.

#### Feature Selection: Comparing Identical Gene Sets

**Figure 5:**
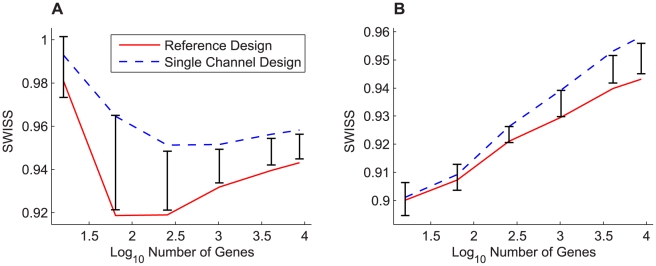
Feature selection: comparing identical gene sets, dataset I. The SWISS scores for the reference design (solid red) and single channel design (dashed blue) along with corresponding 90% confidence intervals (black bars) calculated from the SWISS permutation test are shown for dataset I. The genes for both designs in A were filtered according to variance across all arrays in the single channel design, and the genes in B were filtered according to variance across all arrays in the reference design. The SWISS scores in B are lower than those in A, which suggests that filtering genes using the reference design is better than filtering genes using the single channel design. Also, there are gene filterings in both A and B where there is no significant difference between the single channel and reference designs (both the red and blue lines lie inside the black bars).

Figure 4 compares the SWISS scores of the reference and single channel designs when we filter by gene variance across all arrays. As previously noted, we may have been comparing different gene sets because the most variable genes in the reference design did not necessarily coincide with the most variable single channel design genes. We decided to compare the reference and single channel designs using the same gene sets. Figure 5 shows this comparison for dataset I, along with the corresponding 90% confidence intervals from the permutation test. Figure 5A compares the two designs using the most variable genes from the single channel design, and Figure 5B uses the most variable genes from the reference design. Note that the blue dashed line (single channel design) from Figure 5A and the solid red line (reference design) from Figure 5B are the same lines shown in Figure 4A.

Remember that in Figure 4A, the reference design was always significantly better than the single channel design. However, in Figures 5A and 5B, there are gene filterings where there is no significant difference between the two designs. This is especially apparent in Figure 5B, where there is no significant difference between the reference and single channel designs until at least 1000 genes are included. Also, both the red and blue curves in Figures 5A and 5B have very similar shapes, as opposed to Figure 4A where the curves had different shapes. From this, we conclude that when comparing two different gene filterings, the SWISS curves may have very different shapes (as in Figure 4A). However, if we use the same gene sets on both curves, the SWISS curves should have very similar shapes (as in Figure 5).

When comparing Figure 5A with 5B, notice that the SWISS scores in 5B are always less than or equal to the SWISS scores in 5A. This implies that the filtering method determined by choosing the most variable genes in the reference design is superior to choosing the most variable genes in the single channel design. We conclude that for dataset I, we need the reference design when filtering genes by variation across arrays, but once we have selected the gene set, the single channel design is statistically equivalent to the reference design for up to 1000 genes.

### Experimental Application II: Affymetrix versus Agilent Microarray Platforms

#### Comparison of Affymetrix Pre-Processing Methods

Similar to comparing different normalizations of the single channel design in the first experimental application, we use SWISS to compare two different Affymetrix pre-processing methods: RMA and the Affymetrix Micro Array Suite 5.0 (MAS 5.0). Arrays from test site 1 (n = 20) were analyzed. This eliminated the need for any intrasite adjustment. The SWISS scores of RMA and MAS 5.0 processed data are 0.06 and 0.48, respectively. The difference between these scores is statistically significant. This result is consistent with the results of Millenaar *et al* [47] who conclude that after comparing six different algorithms (including MAS 5.0), RMA gave the most reproducible results.

#### Evaluating the Effect of Affymetrix and Agilent Intersite Reproducibility

We now use SWISS to compare the intersite reproducibility of the Affymetrix, Agilent one-color, and Agilent two-color microarray platforms (datasets V – VII). For this example, a low value of SWISS would mean that the sample replicates lie very close to each other in *d*-dimensional space. There was only one site (test site 2) common between all three platforms under consideration, the U.S. Food and Drug Administration (FDA), and hence we only consider data from this site. Because only two of the four samples (samples A and B) were assayed on the Agilent two-color microarrays, we only consider all four samples when comparing Affymetrix with Agilent one-color microarrays.

When considering all four samples, Affymetrix has a SWISS score of 0.01, which is significantly lower than the Agilent one-color SWISS score of 0.36. When considering only samples A and B, the SWISS scores are 0.007 for Affymetrix, 0.18 for Agilent one-color, and 0.80 for Agilent two-color. All of the pair wise tests between the three platforms return significant p-values, as shown in Supplementary Figure S1. From this analysis, we can conclude that in the MAQC dataset, Affymetrix platform is the best platform in terms of intersite reproducibility, followed by Agilent one-color, with Agilent two-color platform in third position. In this example, we have confirmed, using objective and hypothesis-driven techniques, the conclusion reached by Shi *et al* [23] using a more qualitative approach.

### Experimental Application III: RNA-Seq versus Gene Expression Microarrays

**Figure 6:**
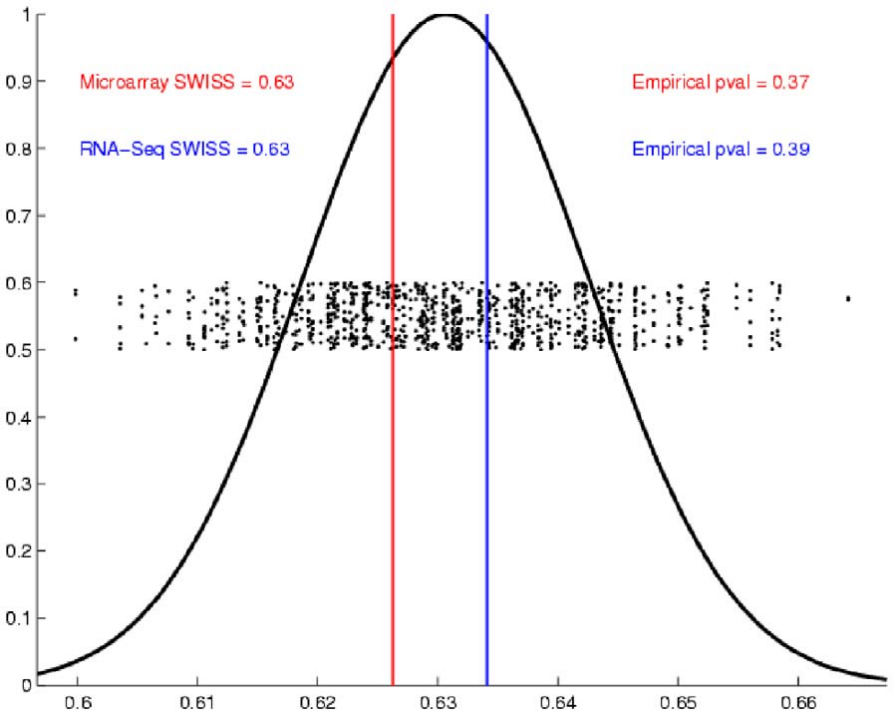
SWISS permutation test results, Experimental Application III. SWISS hypothesis test results for Experimental Application III. Because both p-values are greater than 0.05, we conclude that there is no significant difference between a single Agilent two-color microarray and one lane of RNA-Seq.

For our final application, we use SWISS to compare the performance of a single Agilent two-color array with one lane of RNA-Seq (datasets VIII and IX). Samples were classified by their biological phenotype, either Basal or Luminal. The SWISS scores for RNA-Seq and the microarray data were both 0.63 (as shown in Figure 6). From this analysis, we conclude that there is no significant difference between one lane of RNA-Seq and a single Agilent microarray. This result is consistent with the findings of Marioni *et al* [27], who found one lane of RNA-Seq to be comparable to a single Affymetrix microarray.

## Discussion

We have presented a simple statistical tool, SWISS, which can be used to measure how effectively data are clustered into given phenotypes/classes. Because SWISS is a standardized value, its scores can be compared across different gene spaces. We also presented a permutation-based hypothesis test that reports a p-value which allows investigators to test whether two methods applied to the same dataset are equivalent, or if one method does a better job of clustering the data. SWISS can be used to answer questions related to experimental processing, computational processing, and feature selection.

In the current manuscript we present an initial exploration of three applications to illustrate the potential range of problems for which SWISS might be considered. In Experimental Application I, we used SWISS to address the one-color versus two-color microarray problem on a variety of levels. First, we looked at normalization techniques for single channel data, where SWISS provided a convenient method to compare different normalizations. Second, we showed that SWISS is a convenient tool for comparing different versions of arrays for experimental normalization. Third, we used SWISS to evaluate the impact of feature selection on different platforms. In Experimental Application II, we used SWISS to compare two different microarray platforms, Affymetrix and Agilent, as well as competing Affymetrix pre-processing methods. A particular strength of SWISS's use of standardized Euclidian distance between samples and clusters (noting that other measures may substitute for distance) is that comparisons across platforms are directly interpretable. Competing approaches to SWISS for cross-platform technology comparisons frequently rely on transformations of the data to render competing platforms similar in their component elements. Such decisions such as gene filtering and cross-platform gene annotation may influence the interpretation of the results, as we document in Figure 4. Depending on which set of filtered genes are used, an investigator may reach different conclusions about the superiority of a platform. More troubling, the set of assumptions, such as filtering, may be based on the performance of those genes in one platform over the genes that might have been chosen by the other, ultimately biasing the analysis to favor a potentially spurious result. While SWISS does not necessarily account for the full range of potential biases, it does allow for decisions about data transformations such as gene filtering to be made independently for each data source. Finally, in Experimental Application III, we used SWISS to compare two different gene expression technologies: NextGen sequencing and microarrays, a timely problem with few obvious existing methods in the literature. We showed that one lane of RNA-Seq is statistically equivalent to a single gene expression microarray in terms of how well biological phenotypes cluster together. This observation is fundamentally important as we look to the future of experimental design in this field.

We recognize that the examples we provide cover a wide range of quantitative problems, and that in some cases a competing method might be considered or may have previously been suggested. In general, however, we have found that such competing methods are either limited in the range of problems which they address or subjective in their interpretation. For example, in Experimental Applications II and III, our analyses based on SWISS agreed with results found in the literature, such as Affymetrix having the best intersite reproducibility [23] and one lane of RNA-Seq being comparable to a single gene expression microarray [27]. In order for these authors to make their conclusions, they performed three or more different gene by gene analyses. In contrast, we took a multivariate approach to the problem by using SWISS. For example, Shi *et al* [23] draw their conclusion that Affymetrix has the best intersite reproducibility by comparing

- Coefficient of Variation (CV) of the quantitative signal values between the intrasite replicates,
- Total CV of the quantitative signal, which included both the intrasite repeatability as well as variation due to intersite differences, and
- Percentage of the common genes with concordant detection calls between replicates of the same sample type.

Also, Marioni *et al* [27] draw their conclusion that one lane of RNA-Seq is comparable to a single Affyemetrix array by comparing

- Counts of RNA-Seq with normalized microarray intensities,
- Estimated log_2_ fold changes, and
- Overlap between genes called as differentially expressed.

Most of their analyses relied on subjective cutoffs determined by the author rather than on easily interpretable p-values. Additionally, we are able to draw the same conclusions with only one analysis based on our SWISS method (compared to three each).

While stressing the broad applicability of SWISS to a range of analytical problems and the ease of its use, we would like to acknowledge some important weaknesses as well. First, high-dimensional data such as these array experiments are the product of complex protocols and depend on the quality of reagents and samples. Any change in upstream elements, such as a lab protocol or normalization method, might influence the resulting SWISS scores dramatically. For example, while we showed that the SWISS score of the Affymetrix arrays was significantly lower than the SWISS score of the Agilent arrays, this result is most interpretable in light of the complex set of protocols that generated the MAQC data and might not generalize to other labs or samples. Additionally SWISS scores may not represent the only criterion on which one method is preferred. For example, we showed that RMA gives more reproducible results than MAS 5.0. However, some investigators may prefer using MAS 5.0 because it is more conservative, gives positive output values, down-weights outliers, and minimizes bias [37].

We also acknowledge that while SWISS is convenient to implement across a broad set of analyses, there are likely cases where more dedicated methods would likely provide more nuanced insights. For example, SWISS should not be the only tool used when the investigator is interested in performing an in-depth analysis of competing methods or platforms, such as comparing a new normalization method with other established normalization methods. We should also acknowledge that the examples we have provided should be viewed with extreme caution in terms of the potential to introduce bias. We have shown examples in which one might be tempted to select what appears to be optimal gene sets based on SWISS scores. This is not the intention of the examples but rather to demonstrate the opposite: to document the impact on SWISS scores by varying gene lists. When approached with this caution in mind we feel that these concerns are offset by the broad scope of applicability that our SWISS method offers. Future work will address the concerns about bias adjustment.

We hope investigators will see many uses for SWISS when considering competing processing methods or datasets for evaluating complex multidimensional problems, and will consider incorporating SWISS into their respective pipelines.

## Supporting Information

Text S1 Description of permutation-based SWISS hypothesis test.
(0.05 MB PDF)

Table S1 Consensus clustering results.
(0.03 MB PDF)

Table S2 Effect of clustering samples on SWISS scores for Experimental Application I.
(0.01 MB PDF)

Figure S1 SWISS permutation test results, Affymetrix and Agilent platforms.
(0.06 MB PDF)
