# Supplementary material for: SWISS MADE: Standardized WithIn Class Sum of Squares to Evaluate Methodologies and Dataset Elements
Source: PLoS One. 2010 Mar 26;5(3):e9905. doi: 10.1371/journal.pone.0009905 (PMC2845619; doi:10.1371/journal.pone.0009905)
Supplement: Text S1 — Description of permutation-based SWISS hypothesis test. (0.05 MB PDF) [file pone.0009905.s001.pdf]

Text S1. Description of permutation-based SWISS hypothesis test.

Suppose we apply two different processing methods, say A and B, to the same N experimental samples. Let X_{ij} be a d_1-dimensional vector of covariates of the j^{th}observation ($j = 1, 2, \dots n_i$) from the ith class ($i = 1, 2, \dots K$) from method A, and Y_{ij} be a d_2-dimensional vector of covariates of the j^{th}observation from the ith class from method B. Let X be a $d_1 \,\mathrm{x}\, N$ matrix of the X_{ij}'s, Y a $d_2 \,\mathrm{x}\, N$ matrix of the Y_{ij}'s, and C an N-dimensional vector of class labels corresponding to the columns of X and Y. Let $\bar{X}$ and $\bar{Y}$ be the d_1- and d_2-dimensional overall mean vectors, and $\bar{X}_i$ and $\bar{Y}_i$ be the mean vectors of class i of X and Y, respectively.

1. Calculate the $2 \,\mathrm{x}\, N$ sum of squared deviation matrices D_A and D_B.

$$D_A(1, m) = \sum_{j=1}^{d_1} \left\{ X(j, m) - \bar{X}_{C(m)}(j) \right\}^2$$

$$D_B(1, m) = \sum_{j=1}^{d_2} \left\{ Y(j, m) - \bar{Y}_{C(m)}(j) \right\}^2$$

$$D_A(2, m) = \sum_{j=1}^{d_1} \left\{ X(j, m) - \bar{X}(j) \right\}^2$$

$$D_B(2, m) = \sum_{j=1}^{d_2} \left\{ Y(j, m) - \bar{Y}(j) \right\}^2$$

   where $m = 1, 2, \dots N$. In other words, each column of the squared deviation matrix is the sum of squared deviations of the corresponding sample to its respective class mean (row 1) and overall mean (row 2). Note that summing over the first row gives the Total WithIn class Sum of Squares (Total WISS), and summing over the second row gives the Total Sum of Squares (SST).

2. Standardize each element in D_A and D_B by dividing by the corresponding Total Sum of Squares. That is, for $l \in \{A, B\}$,

$$D_l = \left(\frac{1}{\sum_{m=1}^{N} D_l(2, m)} \right) D_l$$

3. Calculate SWISS scores, the ratio of total within class sum of squares over total sum of squares, for Methods A and B. For $l \in \{A, B\}$,

$$\mathrm{SWISS}_l = \frac{\sum_{m=1}^{N} D_l(1, m)}{\sum_{m=1}^{N} D_l(2, m)} = \sum_{m=1}^{N} D_l(1, m).$$

4. Calculate the permuted population of SWISS scores. Let n_{sim} be the number of permutations. For the analyses in our paper, we set $n_{sim} = 1000$.

   (a) Calculate an $n_{sim} \,\mathrm{x}\, N$ matrix R of random uniform numbers between 0 and 1.

   (b) Calculate the n_{sim}-dimensional vector P of SWISS scores from the permuted population. That is, for permutation $i = 1, 2, \dots n_{sim}$,

$$P(i) = \frac{\sum_{m=1}^{N} I_{R(i,m)<0.5} D_A(1, m) + I_{R(i,m)>0.5} D_B(1, m)}{\sum_{m=1}^{N} I_{R(i,m)<0.5} D_A(2, m) + I_{R(i,m)>0.5} D_B(2, m)}$$

   where

$$I_{R(i,m)<0.5} = \begin{cases} 1 & \text{if } R(i, m) < 0.5 \\ 0 & \text{otherwise} \end{cases}$$

So, for each sample in each permutation, we are randomly choosing which within class and overall sum of squared deviations to use: Method A or Method B. Note that for each permutation, we are not recalculating the mean vectors nor the sum of squared deviations. Thus, it is possible to have a permutation where SST is actually smaller than the Total WISS, and hence, the permuted ratio is larger than 1.

5. Calculate the empirical p-values. For $l \in \{A, B\}$,

$$\text{p-value}_l = \min\left\{\frac{\#\ \text{ of } P(i) < \text{SWISS}_l}{n_{sim}}, 1 - \frac{\#\ \text{ of } P(i) < \text{SWISS}_l}{n_{sim}}\right\}.$$

Two p-values should be reported, one for each method.

We conclude that if both p-values are less than 0.05, that the method with the smaller SWISS score is significantly better at clustering the classes than the other method. If both p-values are not less than 0.05, we conclude that there is no significant difference between Method A and Method B.
