# Supplementary material for: SWISS MADE: Standardized WithIn Class Sum of Squares to Evaluate Methodologies and Dataset Elements
Source: PLoS One. 2010 Mar 26;5(3):e9905. doi: 10.1371/journal.pone.0009905 (PMC2845619; doi:10.1371/journal.pone.0009905)
Supplement: Table S1 — Consensus clustering results. (0.03 MB PDF) [file pone.0009905.s002.pdf]

Dataset I: Single Channel Design

		Clustering 1	Clustering 2
Phenotype	ER +	10 (33%)	20 (67%)
	ER -	2 (22%)	7 (78%)

Dataset I: Reference Design

		Clustering 1	Clustering 2
Phenotype	ER +	10 (33%)	20 (67%)
	ER -	9 (100%)	0

Dataset II: Single Channel Design

		Clustering 1	Clustering 2
Phenotype	ER +	26 (96%)	1 (4%)
	ER -	5 (20%)	20 (80%)

Dataset II: Reference Design

		Clustering 1	Clustering 2
Phenotype	ER +	26 (96%)	1 (4%)
	ER -	5 (20%)	20 (80%)

Dataset III: Single Channel Design

		Clustering 1	Clustering 2
Phenotype	Tumor	7 (88%)	1 (13%)
	Normal	2 (25%)	6 (75%)

Dataset III: Reference Design

		Clustering 1	Clustering 2
Phenotype	Tumor	7 (88%)	1 (13%)
	Normal	2 (25%)	6 (75%)

Contingency tables of biological phenotype and consensus cluster as shown for datasets I-III. Percentages are the proportion of the phenotype in a consensus cluster.
