# Supplementary material for: SWISS MADE: Standardized WithIn Class Sum of Squares to Evaluate Methodologies and Dataset Elements
Source: PLoS One. 2010 Mar 26;5(3):e9905. doi: 10.1371/journal.pone.0009905 (PMC2845619; doi:10.1371/journal.pone.0009905)
Supplement: Table S2 — Effect of clustering samples on SWISS scores for Experimental Application I. (0.01 MB PDF) [file pone.0009905.s003.pdf]

Dataset	No. Genes	Design	Phenotype SWISS	Cluster SWISS	Sig. Diff?
Dataset I	800	Single Channel	0.946	0.896	No
		Reference	0.928	0.913	No
Dataset II	2000	Single Channel	0.886	0.840	Yes
		Reference	0.880	0.821	Yes
Dataset III	4000	Single Channel	0.818	0.712	No
		Reference	0.803	0.674	No

SWISS scores of datasets I – III of Experimental Application I when classifying samples according to their biological phenotype and their Consensus clustering classifications. We also report the number of genes included in the analysis after filtering based on Median Absolute Deviation (MAD), and whether there is a significant difference between the phenotype SWISS and the clustering SWISS scores.
