# Supplementary material for: SWISS MADE: Standardized WithIn Class Sum of Squares to Evaluate Methodologies and Dataset Elements
Source: PLoS One. 2010 Mar 26;5(3):e9905. doi: 10.1371/journal.pone.0009905 (PMC2845619; doi:10.1371/journal.pone.0009905)
Supplement: Figure S1 — SWISS permutation test results, Affymetrix and Agilent platforms. (0.06 MB PDF) [file pone.0009905.s004.pdf]

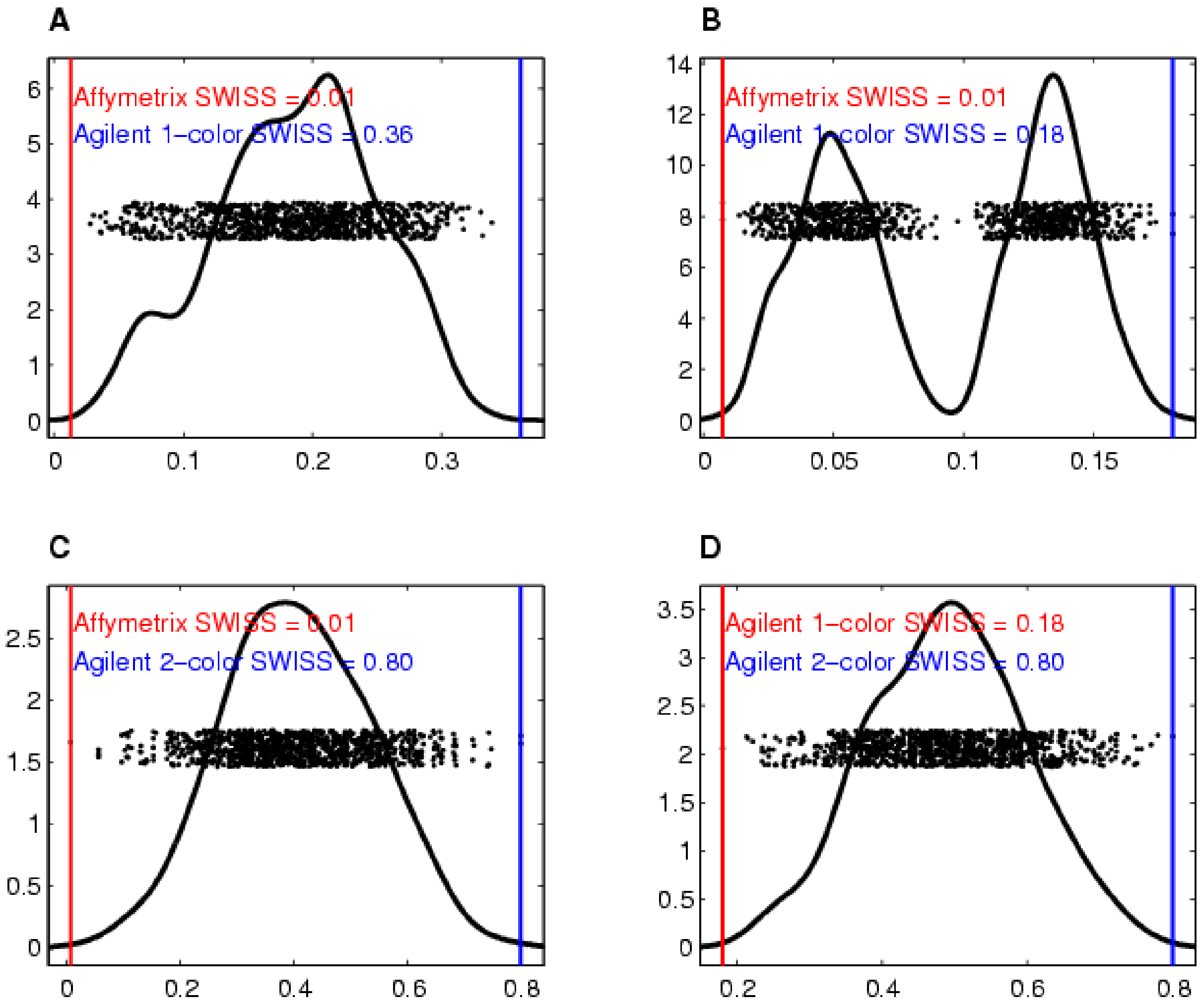

SWISS hypothesis test results for Experimental Application II. Plot A shows Affymetrix versus Agilent one-color using all four samples, and Plot B shows the comparison when only samples A and B are considered. Plots C and D show Agilent two-color versus Affymetrix and Agilent one-color, respectively, when only considering samples A and B. All four tests return p-values of 0, from which we can conclude that the Affymetrix platform outperforms Agilent one-color arrays, and they both outperform Agilent two-color arrays with respect to intersite reproducibility.
